# Macular hole formation, progression, and surgical repair: case series of serial optical coherence tomography and time lapse morphing video study

**DOI:** 10.1186/1471-2415-10-24

**Published:** 2010-09-17

**Authors:** Ronald C Gentile, Gennady Landa, Mauricio E Pons, Dean Eliott, Richard B Rosen

**Affiliations:** 1 Department of Ophthalmology, The New York Eye and Ear Infirmary, New York, NY, USA; 2 New York Medical College, Valhalla, NY, USA; 3 The Doheny Retina Institute, Keck School of Medicine, University of Southern California, Los Angeles, CA, USA

## Abstract

**Background:**

To use a new medium to dynamically visualize serial optical coherence tomography (OCT) scans in order to illustrate and elucidate the pathogenesis of idiopathic macular hole formation, progression, and surgical closure.

**Case Presentations:**

Two patients at the onset of symptoms with early stage macular holes and one patient following repair were followed with serial OCTs. Images centered at the fovea and at the same orientation were digitally exported and morphed into an Audiovisual Interleaving (avi) movie format. Morphing videos from serial OCTs allowed the OCTs to be viewed dynamically. The videos supported anterior-posterior vitreofoveal traction as the initial event in macular hole formation. Progression of the macular hole occurred with increased cystic thickening of the fovea without evidence of further vitreofoveal traction. During cyst formation, the macular hole enlarged as the edges of the hole became elevated from the retinal pigment epithelium (RPE) with an increase in subretinal fluid. Surgical repair of a macular hole revealed initial closure of the macular hole with subsequent reabsorption of the sub-retinal fluid and restoration of the foveal contour.

**Conclusions:**

Morphing videos from serial OCTs are a useful tool and helped illustrate and support anterior-posterior vitreofoveal traction with subsequent retinal hydration as the pathogenesis of idiopathic macular holes.

## Background

Gass and Johnson were the first to classify idiopathic macular holes into four stages using very keen biomicroscopic observations[1-3]. They divided the initial stage into stage 1A, impending macular hole, and stage 1B, an occult hole. Stage 1A has loss of the foveolar depression with a central yellow spot. Stage 1B appears as a yellow ring that is believed to represent centrifugal displacement of the foveolar retina and xanthophyll pigment. Progression to stage 2, or full thickness macular hole, has been reported to occur about fifty percent of the time[2,4-6]. Stage 2 macular holes can be central (with or without a pre-foveolar opacity) or eccentric with a crescent or horseshoe shape break at the edge of the yellow ring. Stage 2 macular holes enlarge and become stage 3 macular holes when they reach a diameter of approximately 400 microns in size. If the posterior vitreous detaches from the optic disc and macula, a stage 3 macular hole becomes a stage 4 macular hole.

Prior to the 1990's, many authors focused significant attention to the pre-foveolar vitreous cortex and its associated traction as playing a primary role in the etiology of idiopathic macular holes[2,3,6-10]. Since then, imaging of the pre-foveolar vitreous cortex has both confirmed and challenged some of their initial clinical impressions[11-23]. New observations and images have added to our understanding of the pathogenesis of macular holes[11-23].

Serial static images are commonly used to explain a dynamic process. In this study, we had the opportunity to use a new medium to illustrate and study idiopathic macular holes. We linked serial optical coherence tomography (OCT) images into a movie format that allowed dynamic visualization of macular holes during formation, progression, and closure.

## Case Presentations

This imaging study adhered to the tenets of the Declaration of Helsinki and The New York Eye and Ear Infirmary Institutional Review Board guidelines. Two patients with early stage macular holes (cases 1 and 2) underwent multiple ophthalmic examinations and OCTs (OCT 2000, Humphrey Instruments, San Leandro, CA) from presentation to surgical repair. An additional patient (case 3) underwent exams and OCTs (OCT 3000, Humphrey Instruments, San Leandro, CA) following surgical repair. Surgery was performed during the first half of the year 2000. OCTs were performed at different meridians through the center of the fovea at each examination. One horizontal scan centered at the fovea (scan length 6 millimeters) at the same location was chosen from each examination and was digitally exported to a motion morphing PC based program called Easy Morph, (Black belt systems, Inc; Glasgow, MT). Four serial OCT images from each patient were used.

The motion morphing software linked the 4 OCT images by streaming progressively morphed images between frames into an Audiovisual Interleaving (avi) movie format. As required by the software, multiple reference points were chosen from one OCT scan to the next in order to ensure a smooth transition during the morphing process and prevent the video from jumping. Twenty-five images between scans were generated and played as a continuous movie using Adobe Premiere 6.0. (Adobe Systems Inc.; San Jose, CA).

### Case 1

**Figure 1:**
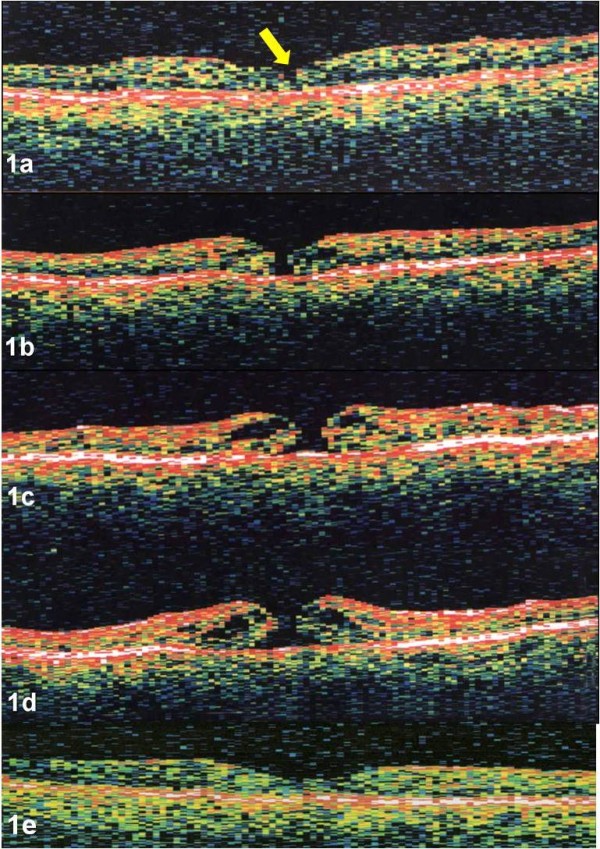
**a-e. Case 1. OCT image of the right eye demonstrated vitreofoveal separation with a small defect in the ILM (yellow arrow) in the center of the fovea (Figure 1a)**. Serial OCTs, performed ten days (Figure 1b), 3 weeks (Figure 1c), and six weeks (Figure 1d) later demonstrated progression to a stage 2 macular hole without evidence of traction. Post-operative OCT revealed closure of the macular hole with restoration of normal foveal contour (Figure 1e). OCTs were performed using OCT 2000, (Humphrey Instruments, San Leandro, CA)

A 70 year-old pseudophakic women presented with an acute central xanthic scotoma and decreased vision in her right eye for two days. Her left eye had a history of non-arteritic ischemic optic neuropathy 2 years prior following cataract surgery. Visual acuities were 20/25 in the right eye and 20/300 in the left eye. Examination of the right eye revealed clear media, blunted foveal reflex, and pre-foveolar operculum. Watzke sign was negative. Examination of the left eye revealed an intraocular lens implant and optic disc pallor. OCT of the right eye demonstrated vitreofoveal separation with a small defect in the internal limiting membrane (ILM) (Figure 1a) in the center of the fovea. The patient had serial ocular examinations with OCTs performed ten days (Figure 1b), 3 weeks (Figure 1c) and six weeks (Figure 1d) following presentation. The hole progressed from a small defect in the ILM to a full thickness stage 2 macular hole with progressive enlargement of the hole. The following movie was created using the above mentioned images only (Movie 1: additional file 1). Visual acuity decreased to 20/50. The patient underwent pars plana vitrectomy with posterior vitreous detachment without additional membrane peeling, and C3F8 gas tamponade with face down positioning. Postoperatively, visual acuity returned to 20/20 and the macular hole closed with return of normal foveal contour (Figure 1e).

### Case 2

**Figure 2:**
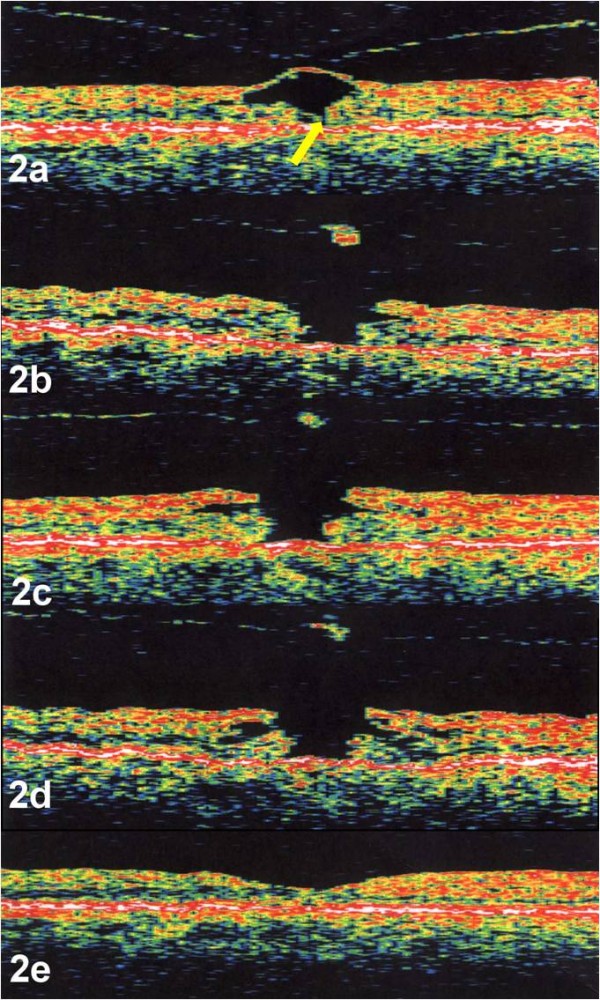
**a-e. Case 2. OCT image of the right eye demonstrated a stage 1 macular hole or cyst with focal attachment of the hyaloid to the fovea**. The yellow arrow designates a discontinuity of the outer fovea (yellow arrow) (Figure 2a). OCT, performed 3 weeks later, revealed avulsion of the inner portion of the foveal cyst with a break in the ILM and progression of the macula hole to a full thickness stage 2 hole with overlying operculum (Figure 2b). Further enlargement of the hole was documented one month (Figure 2c) and two months later (Figure 2d) without evidence of traction. Post-operative OCT revealed closure of the macular hole with restoration of normal foveal contour (Figure 2e). OCTs were performed using OCT 2000, (Humphrey Instruments, San Leandro, CA)

A 50 year-old phakic woman presented with decreased vision in her right eye for two weeks. Visual acuity was 20/50 in the right eye and 20/20 in the left eye. Ophthalmic examination of the right eye revealed a stage 1 macular hole with foveolar elevation. OCT imaged a focal attachment of the hyaloid to the center of the fovea with intact inner foveolar cyst and a tiny defect involving the outer retinal layer (Figure 2a). The left eye was normal. Repeat examinations and OCTs in the right eye remained unchanged until two months later. The right eye had a decrease in visual acuity to 20/200 and OCT revealed progression of the macular hole to a full thickness stage 2 hole with overlying operculum (Figure 2b). Further enlargement of the hole over time was documented one month (Figure 2c) and two months (Figure 2d) later. The following movie was created using the above mentioned images only (Movie 2: additional file 2). The patient underwent pars plana vitrectomy with posterior vitreous detachment, and C3F8 gas tamponade with face down positioning. During the vitrectomy, a Tano Diamond Dusted Membrane Scraper (Synergetics, Inc. O'Fallon, MO) was used to remove a mild cellophane epiretinal membrane around the macular hole. Post-operatively, visual acuity in the right eye improved to 20/20 and OCT revealed normal foveal contour (Figure 2e). The pre-foveal operculum obtained through the vitrector under manual aspiration during surgery underwent histopathological evaluation using electron microscopy and revealed glial elements and a portion of ILM.

### Case 3

**Figure 3:**
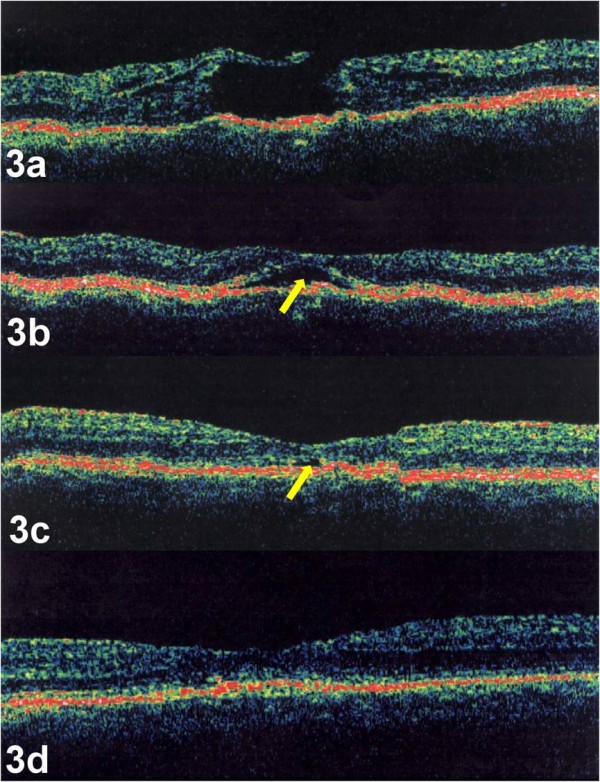
**a-d. Case 3. OCT image of the right eye demonstrated a full thickness stage 2 macular hole with underlying drusen (Figure 3a)**. Post-operative OCT images, performed 2 weeks (Figure 3b), 4 weeks (Figure 3c), and 8 weeks (Figure 3d) after surgery, demonstrated closure of the hole with absorption of the sub-retinal fluid (yellow arrow). OCTs were performed using OCT 3000, (Humphrey Instruments, San Leandro, CA)

A 60 year-old phakic women with a history of macular drusen presented with a decrease in vision in the right eye for 3 months. Visual acuity was 20/70 in the right eye and 20/30 in the left eye. Ophthalmic examination and OCT revealed a full thickness stage 2 macular hole in the right eye with drusen (Figure 3a). The patient underwent a pars plana vitrectomy with posterior vitreous detachment without additional membrane peeling, and SF6 gas tamponade with face down positioning. OCT of the right eye was performed 2 weeks (Figure 3b), 4 weeks (Figure 3c), and 8 weeks (Figure 3d) after surgery. OCT revealed initial closure of the hole with resolution of the cystic changes and persistent subretinal fluid. Postoperatively, total absorption of the sub-retinal fluid occurred. The following movie was created using the above mentioned images only (Movie 3: additional file 3). Visual acuity improved to 20/30.

## Conclusions

Morphing videos from serial OCTs allowed the OCTs to be viewed dynamically and helped illustrate anatomical changes that occurred during idiopathic macular hole formation, progression, and surgical closure. The characteristics of the neurosensory retina and vitreofoveal interface in each OCT image for all three cases are described in Table 1.

**Table 1:** Presence of sub-retinal fluid (SRF), intraretinal cystoid spaces (ICS), and vitreofoveolar traction (VFT) in each OCT image

Case	Figure	SRF	ICS	VFT
**1**	a	no	no	no

	b	no	yes	no

	c	yes	yes	no

	d	yes	yes	no

**2**	a	no	no	yes

	b	no	yes	no

	c	yes	yes	no

	d	yes	yes	no

**3**	a	yes	yes	no

	b	yes	no	no

	c	yes	no	no

	d	no	no	no

The serial OCTs and videos supported anterior-posterior vitreofoveal traction as the initial event in macular hole formation, highlighted by case 2. Although case 1 had no evidence of vitreofoveolar traction and only a small defect in the ILM by OCT, the presence of a pre-foveolar operculum on clinical exam also supports this theory. Progression and enlargement of the macular hole occurred with increased cystic thickening of the fovea without evidence of further vitreofoveal traction. The initial event involving a break in the foveolar ILM was followed by destabilization of the underlying neurosensory retina with cystic formation within the outer plexiform layer. During cystic formation, the macular hole enlarged as the edges of the hole became elevated from the retinal pigment epithelium (RPE) with an increase in subretinal fluid, highlighted by both case 1 and 2. Surgical repair of a macular hole revealed both initial closure of the macular hole and resolution of the intraretinal cytoid spaces with subsequent reabsorption of the sub-retinal fluid and restoration of the foveal contour, highlighted by case 3.

OCT has improved our ability to diagnose macular holes[14,19]. Correlations between clinical and OCT findings have expanded our understanding of the pathogenesis of idiopathic macular holes and new theories have been postulated as imaging devices have improved[11-18,21-23]. Vitreoretinal tractional forces have been shown to be responsible for macular hole formation in the majority of cases; however, there are cases that do not conform to the current theories[24-28]. These cases present a challenge, as gaps in our understanding of macular hole formation and progression still exist.

We have applied a new medium to ophthalmic imaging, called morphing, to dynamically visualize serial OCT scans. Morphing is a visual effect that transitions one image into another image. Prior to the 1990s, morphing was primarily used to produce special effects in motion pictures and animations. As computer capabilities and software improved, morphing has been used in a variety of scientific specialties including biology, paleontology, anthropology, plastic surgery, and medical education[29-34]. Although morphing has been used as part of film festival presentations at the American Society of Retina Specialists (Gentile and Ponce 2005, Nawrocki et al 2008, Nawrocki et al 2009) and as additional supporting information in online versions of articles [18], the technique remains unpublished in the field of ophthalmology. This process has allowed us to link serial static images and illustrate the pathogenesis of idiopathic macular hole formation, progression, and surgical closure.

The pathogenesis of idiopathic macular holes can be divided into phases. The first phase, or formation phase, includes the initiating event. This phase is followed by a pivotal event that determines if the macular hole will enter the second phase, or progression phase, and become a clinically visible full thickness macular hole. A third phase, or closure phase, includes the process by which the macular hole closes; most often by successful surgical repair and rarely by spontaneous closure. This study, in conjunction with other studies helps elucidate and illustrate the three phases and the pivotal event leading to an idiopathic macular hole.

The first phase, or initiating event has been shown to include a posterior hyaloid detachment from the fovea with foveolar dehiscence[4,6-10,12,13,15,16]. Serial optical coherence tomographs and morphing videos confirm this concept. In both case 1 and 2, anterior vitreofoveolar traction created a defect in the ILM. This was especially highlighted in the morphing video of case 2. This process has been shown by others using dynamic B-scan[6].

The second phase, or progression phase, occurred without evidence of additional traction in our cases. Serial optical coherence tomographs and morphing videos support the hydration theory of macular hole progression, describe by Tornambe in 2003[23]. Our first patient presented with a foveal vitreous detachment and pre-foveolar operculum, seen on first examination with a small defect in the ILM. The unusually early presentation of case 1, within 2 days of symptoms, may have been due to the patient's monocular status. As vitreous fluid entered through the ILM defect, hydration of the outer neurosensory retina occurred with separation of its edges and expansion of the hole in the absence of further vitreous traction. Tangential traction in our opinion was not observed. None of our patients presented with a significant epiretinal membrane that could have contracted and expanded the hole. The finite elasticity of the ILM cannot be ruled out; however we would have expected a more rapid progression of the hole if this was a predominate force. The non-membranous layers of the foveola rely on an intact and healthy Müller cell layer to bond them together, and most likely they cannot prevent macular hole formation once the ILM and external limiting membrane (ELM) are breached and cystoid retinal hydration begins.

Surgical repair of a macular hole is believed to result from a plug of glial tissue bridging the hole[35]. As seen in case 3, closing the communication between the photoreceptor layer and the vitreous cavity permitted dehydration of the cystic retinal changes first. This allowed the RPE to pump out sub-retinal fluid and for the fovea to regain normal contour. The delay in reabsorbing sub-retinal fluid compared to other studies[36] may have been related to the drusen and associated limited RPE pumping potential in case 3.

The authors postulate, based on this study and others that the pathogenesis of an idiopathic macular hole is as follows: The first phase or initiating event begins with vitreofoveal separation with adherence of the foveolar ILM to the posterior vitreous cortex. As anterior-posterior traction occurs, the hyaloid can either remain attached to the foveolar and result in an intraretinal cyst, or stage 1 macular hole as in our case 2, or completely detach from the fovea as in our case 1. Forces transmitted to the Müller cell cone[37] can disrupt its structural support of the foveolar photoreceptors. Disruption of both the ILM and ELM becomes the pivotal event that determines if a full thickness macular hole will develop. Disruption of both membranes can occur simultaneously, as in Case 1, or involve the ELM before the ILM as in Case 2 and form what has been referred to as an outer lamellar hole[22]. If the ELM does not become breached and the foveolar cortical hyaloid detaches, the stage 1 macular hole becomes an aborted macular hole. Although rare, an aborted macular hole can later progress to a full thickness macular hole if the ELM becomes disrupted from tangential epiretinal proliferation[26,27].

As in our cases, the second phase or progression phase, can occur without traction. Once both the ILM and ELM becomes breached, disruption of the seal between the neurosensory retina and the RPE pump causes hydration of the foveolar with progressive enlargement of the hole. Hydration of the perifoveolar retina with cystic changes results in elevation of the edges of the hole and subsequent enlargement of the macular hole. Successful repair of a macular hole requires adjacent migratory glial cells to bridge the hole and reestablish the seal between the neurosensory retina and the RPE pump. This allows dehydration of the cystic retina first followed by reabsorption of the subretinal fluid as seen in case 3.

Limitations of our study include the few number of patients studied and the use of an earlier generation OCT. Performing additional analysis of serial OCT scans with higher resolution spectral domain OCT images will be helpful to further substantiate and elucidate this process and our hypothesis since different retinal layers, including the ILM and ELM, can be better identified with this technology. In addition, having more OCT images over time would prevent potential morphing artifacts.

In summary, serial OCTs with corresponding morphing videos provide a new modality to visualize macular holes dynamically and support anterior posterior vitreofoveal traction with a break in the ILM as the initiating event in idiopathic macular hole formation. The progression of the macular hole appears to result from destabilization of the underlying retina with progressive hydration. This is consistent with the hydration theory of macular hole progression of Dr. Tornambe[23]. Surgically, closure of the macular hole appears to occur prior to restoration normal foveal contour. The methods used in the present study add a new tool to help understand the process of macular hole formation, progression, and surgical closure.

## Consent

Written consent was obtained from the patients for publication of this study.

## Competing interests

RCG, GL, MEP, DE, none; RBR is a consultant for OPKO, Clarity and Allergan.

## Authors' contributions

RCG conceived of the study, designed, analyzed the data, interpreted the data and drafted the manuscript; GL helped in interpretation of the data and drafted the manuscript; MEP helped in interpretation of the data and drafted the manuscript; DE and RBR were involved in drafting the manuscript and revising it critically for important intellectual content. All authors read and approved the final manuscript.

## Pre-publication history

The pre-publication history for this paper can be accessed here:

http://www.biomedcentral.com/1471-2415/10/24/prepub

## Supplementary Material

Additional file 1 **Morphing video file for case 1**. The video demonstrates correspondent serial OCTs (4 OCTs over 6 weeks). Click here for file

Additional file 2 **Morphing video file for case 2**. The video demonstrates correspondent serial OCTs (4 OCT's over 11 weeks). Click here for file

Additional file 3 **Morphing video file for case 3**. The video demonstrates correspondent serial OCTs (4 OCTs over 10 weeks). Click here for file
